# Unlabeled-Data-Enhanced Tool Remaining Useful Life Prediction Based on Graph Neural Network

**DOI:** 10.3390/s25134068

**Published:** 2025-06-30

**Authors:** Dingli Guo, Honggen Zhou, Li Sun, Guochao Li

**Affiliations:** School of Mechanical Engineering, Jiangsu University of Science and Technology, Zhenjiang 212000, China; guodingli990929@163.com (D.G.); zhouhonggen_just@126.com (H.Z.); liguochao@just.edu.cn (G.L.)

**Keywords:** tool RUL prediction, unlabeled data enhancement, graph neural network, transfer learning, multi-sensor data fusion

## Abstract

Remaining useful life (RUL) prediction of cutting tools plays an important role in modern manufacturing because it provides the criterion used in decisions to replace worn cutting tools just in time so that machining deficiency and unnecessary costs will be restrained. However, the performance of existing deep learning algorithms is limited due to the smaller quantity and low quality of labeled training datasets, because it is costly and time-consuming to build such datasets. A large amount of unlabeled data in practical machining processes is underutilized. To solve this issue, an unlabeled-data-enhanced tool RUL prediction method is proposed to make full use of the abundant accessible unlabeled data. This paper proposes a novel and effective method for utilizing unlabeled data. This paper defines a custom criterion and loss function to train on unlabeled data, thereby utilizing the valuable information contained in these unlabeled data for tool RUL prediction. The physical rule that tool wear increases with the increasing number of cuts is employed to learn knowledge crucial for tool RUL prediction from unlabeled data. Model parameters trained on unlabeled data contain this knowledge. This paper then transfers the parameters through transfer learning to another model based on labeled data for tool RUL prediction, thus completing unlabeled data enhancement. Since multiple sensors are frequently used to simultaneously collect cutting data, this paper uses a graph neural network (GNN) for multi-sensor data fusion, extracting more useful information from the data to improve unlabeled data enhancement. Through multiple sets of comparative experiments and validation, the proposed method effectively enhances the accuracy and generalization capability of the RUL prediction model for cutting tools by utilizing unlabeled data.

## 1. Introduction

Statistics show that downtime due to tool wear and damage accounts for approximately 20% of total production time [1]. Furthermore, relevant research shows [2] that only 50% to 80% of tool useful life is utilized efficiently. It is not only necessary to replace tools before they become blunt but also to fully utilize the RUL of tools to avoid resource waste and additional cutting costs. As a result, tool RUL prediction during the process of mechanical cutting is a necessary method. Tool RUL prediction usually depends on tool wear measured during machining to evaluate a tool’s degradation and estimate its residual lifespan.

Tool RUL prediction mainly includes physics-driven and data-driven methods. Li et al. [3] introduced a physics-informed meta-learning framework that employs physical modeling strategies to predict tool wear across varying wear rates. Karandikar et al. [4] developed a physics-guided logistic classification method using logarithmic transformation of inputs to model the nonlinear relationship between cutting speed and tool life for the purposes of tool life modeling and cutting parameter optimization. Physics-driven methods require deep domain expertise and complex mathematical formulations, which can limit their adaptability to different tools and operating conditions, resulting in poor generalization capability.

Data-driven methods mainly include machine learning, especially deep learning methods. Sensors collect signals during the cutting process. Feature vectors are then extracted from these signals and input into machine learning or deep learning algorithms to predict tool RUL. Traditional machine learning methods include principal component analysis (PCA), signal processing, and classifiers [5,6,7,8,9,10]. Deep learning methods include convolutional neural networks (CNNs) [11,12], recurrent neural networks (RNNs) [13,14], autoencoders (AEs) [15], attention mechanisms [16], and others. Deep learning methods offer advantages like automatic feature extraction, the handling of complex nonlinear relationships, the processing of large datasets, robust generalization capability, and end-to-end learning, leading to superior performance in tool RUL prediction.

The research studies mentioned above on predicting tool wear and RUL with deep learning methods were mostly conducted on labeled data. However, in practical cutting processes, labeled data are scarce because accurately measuring tool wear and labeling the corresponding cutting data is a costly and time-consuming task, resulting in limited labeled data and abundant unlabeled data. This unlabeled data also contains valuable information about tool condition and wear, making the effective utilization of unlabeled data crucial for predicting tool RUL. Some researchers have utilized unlabeled data to enhance the performance of their tool wear and RUL prediction models. Shu et al. [17] introduced a self-supervised contrastive learning framework that uses multi-view contrastive learning to predict tool wear effectively with only a few labeled samples. Wang et al. [18] developed a physics-guided neural network that integrates physical knowledge with data-driven techniques, reducing reliance on abundant labeled data. Sun et al. [19] proposed a semi-supervised method using unreliable pseudo-labels, enhancing model generalization capability when there is a lack of reliable annotations. Warke et al. [20] and Zhu et al. [21] designed a hybrid physics–data-driven model fusion framework and an unsupervised dual-regression domain adversarial network, respectively, to adapt to tool wear prediction across varying working conditions, thereby improving robustness and generalization capability on unseen data. Li et al. [22] employed a semi-supervised multi-source meta-domain generalization method with instance-based domain adaptation to address tool wear state prediction under changing cutting conditions. Liu et al. [23] proposed an unsupervised chatter detection method combining autoencoders with a Gaussian mixture model (GMM) and K-means clustering, enabling effective detection without requiring abundant labeled data. In summary, these research studies show that the effective use of unlabeled data can enhance the accuracy and generalization capability of models for tool wear and RUL prediction.

Although these research studies use unlabeled data to varied degrees, limitations remain. Some rely on predefined physical models or domain-specific knowledge, limiting their flexibility and broad applicability across different contexts. Others, while using self-supervised or semi-supervised methods, may require substantial computational resources and time to process large-scale unlabeled data, resulting in inefficiencies. Moreover, several methods may be sensitive to initial parameters or fail to maintain stable performance in complex and variable working conditions. Since tool wear increases with increasing cuts, this paper captures the latent feature in cutting signals that evolves with increasing cuts. This paper defines a criterion to describe the trend of this evolving feature and then trains models on unlabeled datasets to match outputs with the defined criterion. To train for tool RUL prediction using labeled data, this paper first transfers trained parameters from an unlabeled data model via transfer learning, thereby transferring the knowledge learned from unlabeled data. This method is simpler, more efficient, and more generalizable, aiming to significantly enhance the accuracy and robustness of tool RUL prediction by fully utilizing abundant unlabeled data.

Multiple sensors are often used to collect signals during cutting tool operations, and the fusion of multi-sensor data is important to improve unlabeled data enhancement (better suited for the purpose to fully utilize unlabeled data) for tool RUL prediction. CNNs and their variants [24,25,26,27,28,29] have been widely used in the fusion of multi-sensor data for efficient tool RUL prediction as well as certain other models [30,31,32]. However, CNNs are primarily suited for regular grid-like data structures, such as 2D images, and have limited capability in processing non-Euclidean data like complex sensor networks. In multi-sensor data fusion, different types of sensor data often have complex topological relationships and dependencies, which CNNs may overlook by focusing on local spatial information extraction. To extract more valuable information from multi-sensor data for unlabeled-data-enhanced tool RUL prediction, this paper proposes the use of graph neural networks (GNNs) to aggregate multi-sensor signal features. GNNs have been successfully applied in various industrial research areas [33,34,35,36] with excellent performance. GNNs can process non-Euclidean data directly and effectively capture complex dependencies between sensors, making them suitable for the fusion of heterogeneous information from multi-sensor data.

In summary, this paper proposes an unlabeled-data-enhanced tool RUL prediction method based on GNNs. The main contributions of this paper are as follows:

(1) To better achieve unlabeled data enhancement, this paper innovatively introduces a GNN to multi-sensor data fusion for tool RUL prediction. The GNN can directly process non-Euclidean data and capture complex dependencies between sensors, thus extracting more information from the intricate spatial and dynamic correlations in multi-sensor data.
(2) By training on unlabeled data, this paper defines a criterion and loss function to learn knowledge crucial for tool RUL prediction. Model parameters trained on unlabeled data contain the knowledge and are then transferred to another model based on labeled data for tool RUL prediction, completing the unlabeled data enhancement.

The remainder of this paper is organized as follows: Section 2 details the proposed method; Section 3 outlines the experimental design; Section 4 provides results and discussion, validating the method; and Section 5 concludes this paper.

## 2. Methodology

To complete unlabeled-data-enhanced tool RUL prediction, this method primarily consists of the following aspects.

**Figure 1:**
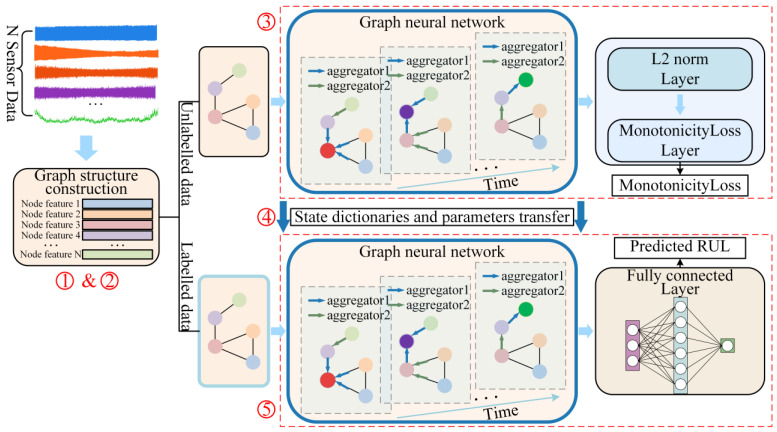
Overall methodology: ➀ Cut signal feature extraction. ➁ Graph-structured data construction. ➂ Use of unlabeled data for ULDTM training. ➃ Parameter transfer. ➄ LDTM outputs the predicted tool RUL of labeled data, followed by training and validating.

Firstly, feature extraction is applied on multi-sensor signals collected during each cut, converting each sensor signal into a fixed-dimensional feature vector. Secondly, given that graph neural networks (GNNs) require graph-structured data as input, the data from a cut is constructed into a graph. These graphs can be classified as either unlabeled or labeled data. This paper constructs 2 models: the ULDTM (unlabeled-data-trained model) and LDTM (labeled-data-trained model). Thirdly, the unlabeled data is input into the ULDTM for training based on the defined criterion and a custom loss function. After training on unlabeled data, parameters of the ULDTM learn certain knowledge crucial for enhancing tool RUL prediction. The labeled data is input into the LDTM for tool RUL prediction. Fourthly, the parameters from the GNN part of the ULDTM are transferred via transfer learning to initialize the GNN parameters in the LDTM, thereby transferring the knowledge learned from the unlabeled data. Finally, the LDTM outputs the predicted tool RUL and uses this output for training and validation. The overall methodology is illustrated in Figure 1.

### 2.1. Feature Extraction

Tool cutting data typically consist of multi-sensor time series (signals). We use feature extraction to convert the time series into fixed-dimensional feature vectors. This paper uses 30 time–frequency-domain characteristics to extract features from time series collected during the tool cutting process.

This paper uses 16 time–frequency-domain characteristics from reference [37]. In addition to the 16 time–frequency-domain characteristics mentioned in the reference, this paper supplemented 14 more time–frequency-domain characteristics. A total of 30 time–frequency-domain characteristics were extracted from the sensor signals, comprising 17 time-domain characteristics and 13 frequency-domain characteristics. In addition to the 16 characteristics mentioned in the citation, the remaining characteristics are as follows.

For a single sample, the collected signal is denoted as *x* = [*x*(1), …, *x*(*i*), …, *x*(*N*)], where *N* represents the number of sampling points in that sample. The time-domain characteristics are calculated using the formulas in Table 1.

To calculate frequency-domain characteristics from multi-sensor time series, this paper uses the Fast Fourier Transform (FFT). Let *s*(*k*) be the spectrum of the signal x, where *k* = 1, 2, …, *K* and *K* is the number of spectral lines. The frequency value of the *k*-th spectral line is denoted as *f*(*k*). The frequency-domain characteristics are calculated using the following formulas in Table 2.

### 2.2. Graph Neural Network

To improve unlabeled data enhancement, this paper uses a GNN for multi-sensor data fusion. The tool cutting process usually employs multiple sensors to collect signals (time series). Using the feature extraction methods described above, each sensor’s signal collected during a single cutting process is converted to a 30-dimensional feature vector. If n sensors are used, a single cutting process signal is converted into n 30-dimensional feature vectors.

In multi-sensor signals, different sensors capture different information, and their data is complexly interrelated. To effectively characterize these relationships and aggregate multi-sensor signal feature vectors for predicting tool RUL, this paper constructs graph-structured data from the multi-sensor network. This graph structure naturally captures the complex dependencies between sensors.

**Graph Construction**

- **Node:** Each sensor is viewed as a node in the graph.
- **Node Embedding:** The 30-dimensional feature vector extracted from each sensor’s signal is used to embed the corresponding node.
- **Edges:** Edges between nodes represent the dependencies or correlations among sensors.

**Figure 2:**
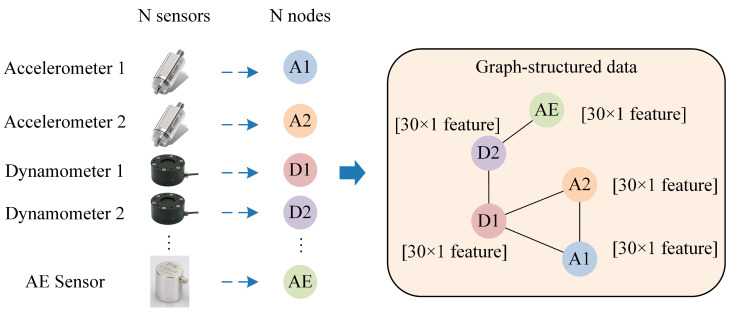
Graph-structured data construction.

Thus, a single cutting process signal is converted into a graph, which consists of nodes with 30-dimensional feature vectors as node embeddings and edges between nodes. This conversion enables a more comprehensive and accurate representation of multi-sensor signals, enhancing effective tool RUL prediction. This method takes advantage of the inherent complexity of multi-sensor signals, providing a robust data framework for tool RUL prediction. The conversion of a single cutting process signal into graph data is illustrated in Figure 2.

Graph neural networks (GNNs) are deep learning models that process graph-structured data. GNNs can capture the complex interactions between sensor nodes using graph convolutional layers, combining signals from different sensors into a unified framework and accomplishing multi-sensor signal feature vector fusion. By more effectively aggregating information from multi-sensor data compared to other deep learning methods, the GNN improves unlabeled data enhancement as well as the accuracy of tool RUL prediction.

**Figure 3:**
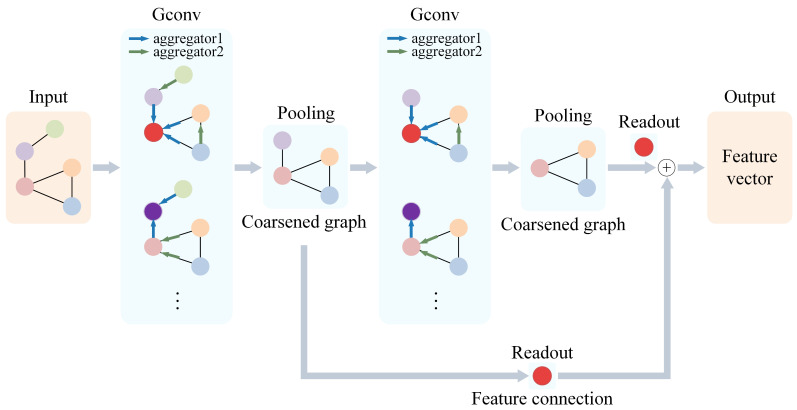
Structure and data processing of GNN.

In the GNN, each node’s representation is updated by aggregating node embeddings from its neighboring nodes using graph convolutional layers. This aggregation process can be performed iteratively, allowing nodes to capture information from neighbors at longer distances. The GNN takes node-embedding vectors and edge indices of a graph as input and returns a single feature vector that represents the entire graph. Specifically, the structure of the GNN is illustrated in Figure 3.

**Graph convolution layers:** These layers work as follows. They input node embeddings and the edge index of a graph. They aggregate node embeddings through 2 layers of graph convolutions (GConv1 and GConv2), followed by batch normalization (BatchNorm). Selecting 2 layers of graph convolutions allows for effective information extraction during node-embedding aggregation and updating, enhancing model accuracy. The 2-layer graph convolution also ensures the model’s running time does not increase significantly, maintaining efficiency. If using GraphSAGE as a graph convolution layer, the process of aggregating node embeddings in GraphSAGE is as follows:

Input: Graph G=(V,E), where *V* is the set of nodes and *E* is the set of edges. Input node embeddings for each node $\left\{x_{v}|v\in V\right\}$; search depth *K*; weight matrices $W_{k},\;k=1,\ldots ,K$; activation function σ; differentiable aggregation functions ${\mathrm{AGGREGATE}}_{k},\;k=1,\ldots ,K$; neighbor selection function N(v), which returns the set of neighboring nodes of node *v*.

The output is the updated node embedding $z_{v}$ for each node *v*.

The algorithm steps are as follows: Initialize the node embedding for each node:

(1) $$h_{v}^{0}=x_{v},\;\forall v\in V$$

GraphSAGE offers several different aggregation function options, which can capture the local features of neighboring nodes and send them to the target node. Commonly used aggregation functions include the following: mean aggregator, pooling aggregator, and LSTM aggregator. For each depth k=1,…,K and each node *v*, aggregate the node embeddings from its neighbors:

(2) $$h_{N(v)}^{k}={\mathrm{AGGREGATE}}_{k}\left(\left\{h_{u}^{k-1}|u\in N(v)\right\}\right)$$

Update the node embedding of node *v* by combining its own information and that of its neighbors:

(3) $$h_{v}^{k}=\sigma \left(W_{k}\cdot \mathrm{CONCAT}(h_{v}^{k-1},h_{N(v)}^{k})\right)$$

$h_{v}^{k}$ represents the representation of node *v* at layer *k*. In each layer, the new node embedding $h_{v}^{k+1}$ of the target node *v* is generated by combining its own embedding $h_{v}^{k}$ with the aggregated embeddings of its neighbors. The specific update rule is usually as follows:

(4) $$h_{v}^{k+1}=\sigma \left(W_{v}^{k}\cdot h_{v}^{k}+W_{N}^{k}\cdot \mathrm{Aggregate}(\{h_{u}^{k},\forall u\in N(v)\})\right)$$

Here, $W_{v}^{k}$ and $W_{N}^{k}$ are learnable weight matrices, and Aggregate is the aggregation function for the features of neighboring nodes. This process is iterated over multiple layers, with each layer further aggregating the local and neighbor information of the nodes.

Normalize the node embedding:

(5) $$h_{v}^{k}=\frac{h_{v}^{k}}{\|h_{v}^{k}{\|}_{2}},\forall v\in V$$

Output the final node embedding for each node:

(6) $$z_{v}=h_{v}^{K},\forall v\in V$$

**Activation function:** Use the ReLU activation function to introduce nonlinearity.

**Graph pooling layer:** After each graph convolution, use a graph pooling layer (PoolLayer) to downsample the graph structure and reduce the number of nodes. After the graph pooling layer, use global mean pooling to convert all node embeddings into a single readout vector (output).

The proposed method using a GNN for multi-sensor data fusion and unlabeled-data-enhanced tool RUL prediction offers a robust and efficient solution for processing complex and interrelated data in practical cutting.

In this paper, the learning rate is set to 0.001 with an Adam optimizer, and a step learning rate scheduler is employed to adjust the learning rate during training. The batch size is fixed at 256 for the training process. The GNN architecture, specifically a GraphSage model, consists of two graph convolutional layers with 1024 hidden dimensions each.

### 2.3. Unlabeled-Data-Trained Model Training

To train a model for capturing latent features from unlabeled data, each unlabeled graph is input into a GNN, which processes the input and outputs an output vector. The GNN outputs *B* output vectors for a batch size of *B* unlabeled graphs. The L2 norms of the output vectors are then calculated. These output vectors subsequently pass through the L2 Norm Layer to obtain their corresponding L2 norms, denoted as *l*_1_, *l*_2_, …, *l*_B_. The L2 norm *l* of an output vector ***v*** *=* (*v*_1_, *v*_2_, …, *v_n_*) is calculated using the following formula:

(7) $$l={\left\|v\right\|}_{2}=\sqrt{\sum_{i=1}^{n}v_{i}^{2}}$$

This paper constructs a norm vector ***L*** *=* (*l*_1_, *l*_2_, …, *l_B_*) using L2 norms for each graph in a batch to calculate the monotonicity loss. Based on the fact that tool wear increases with the number of cuts increasing, we define a criterion that the L2 norm should increase with the cuts increasing. To meet this criterion, we define a monotonicity loss. This norm vector ***L*** is then input into a custom MonotonicityLoss layer, which calculates the monotonicity loss over the elements of L. In this paper, the monotonicity loss for ***L*** elements is defined as follows: for a norm vector, if the preceding element is greater than the succeeding one (i.e., it does not follow monotonically increasing order), the absolute difference between these two elements is calculated and added to the monotonicity loss. Finally, the monotonicity loss is the sum of all such losses. The formula for calculating the monotonicity loss is given by

(8) $$\mathrm{MonotonicityLoss}=\sum_{i=1}^{B-1}\left|l_{i}-l_{i+1}\right|\delta (l_{i}>l_{i+1})$$

**Figure 4:**
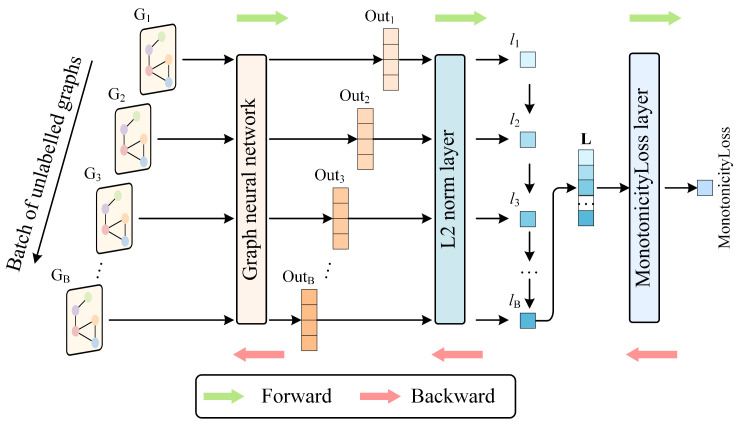
Structure and training process of ULDTM.

To minimize the monotonicity loss, backpropagation is used to gradually reduce its value. A smaller monotonicity loss shows that elements in the norm vector ***L*** are increasing more monotonically, which suggests that the GNN is efficiently capturing latent features from unlabeled tool cutting data. The latent features evolve as the number of cuts increases. The forward and backward propagation processes for unlabeled data through the ULDTM are illustrated in Figure 4.

### 2.4. Tool RUL Prediction and Transfer Learning

Labeled data is used for tool RUL prediction. The labeled dataset is divided into training and validation sets. Specifically, the labeled data is input into the GNN and processed to output vectors (the same as in the ULDTM). These output vectors then pass through a fully connected layer, resulting in a single value that represents the predicted RUL of the tool.

**Figure 5:**
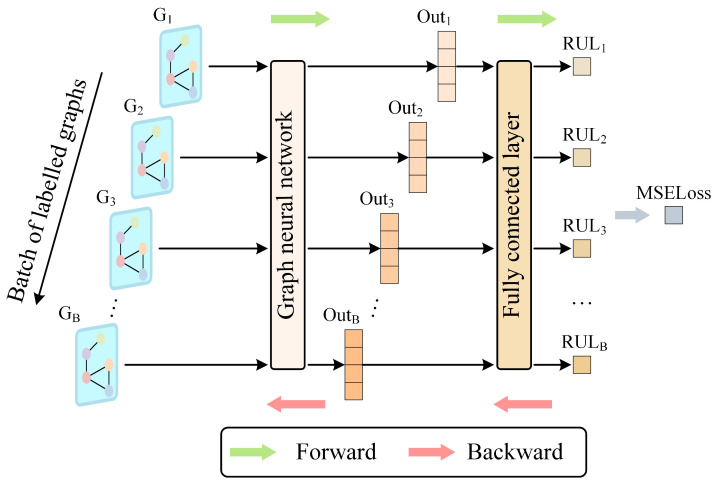
Structure and training and validation processes of LDTM.

The predicted RUL is compared to the actual labels to calculate the mean squared error loss (MSELoss), which is then backpropagated to update the network parameters. The LDTM is structured as shown in Figure 5.

During the unlabeled-data-training process, state dictionaries (model parameters) of the GNN part of the ULDTM are saved at each epoch. During the training process on unlabeled data, an epoch with a smaller monotonicity loss is chosen. The state dictionaries (model parameters) of the GNN part from this epoch are used as the initial parameters for the GNN part of the LDTM, completing transfer learning. Labeled data is then input into the LDTM to predict tool RUL, train, and validate.

**Figure 6:**
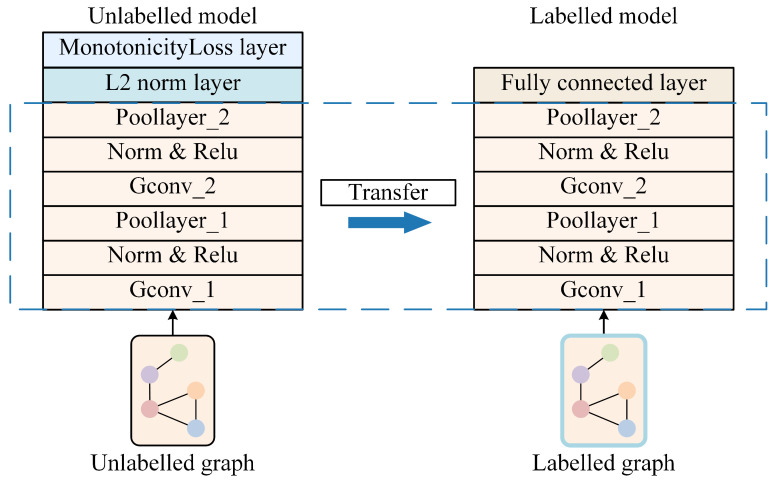
Comparison between the two models and transfer learning.

The GNN parts of both the ULDTM and LDTM are identical. The part of the GNN used in the ULDTM is followed by a monotonicity loss layer to evaluate the model’s capability to capture latent features throughout the cutting process, while the part of the GNN used in the LDTM is followed by a fully connected layer for tool RUL prediction. The comparison between the two models and transfer learning can be described as shown in Figure 6.

## 3. Experimental Design

This paper uses the PHM2010 dataset to predict tool RUL of a three-flute ball nose cutter under identical cutting conditions. Additionally, the JUST_T05 dataset is used as a supplementary experiment.

### 3.1. Experimental Design of PHM2010

**Figure 7:**
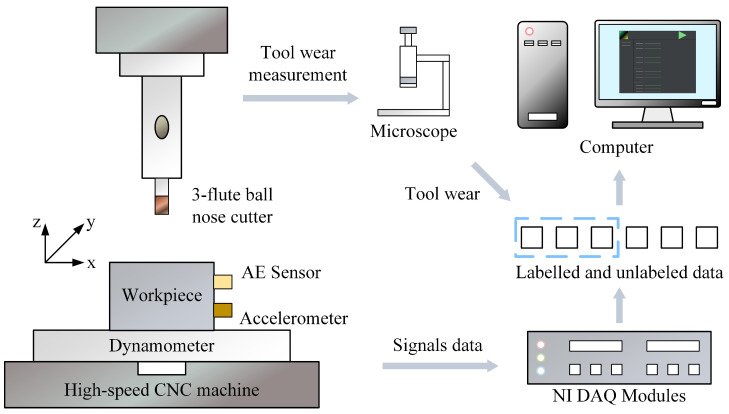
Schematic diagram of the PHM2010 experiment.

The structure of the PHM2010 experiment is shown in Figure 7.

The PHM2010 dataset includes cutting experiments for six tools, denoted as C1 to C6, with each experiment including 315 cuts. The experiments C2, C3, and C5 are unlabeled, while C1, C4, and C6 are labeled. Each dataset contains data collected by seven sensors: three for measuring cutting forces along the X, Y, and Z axes, three for vibration signals along the X, Y, and Z axes, and one for acoustic emission signals. The structure of the PHM2010 dataset is shown in Table 3.

**Figure 8:**
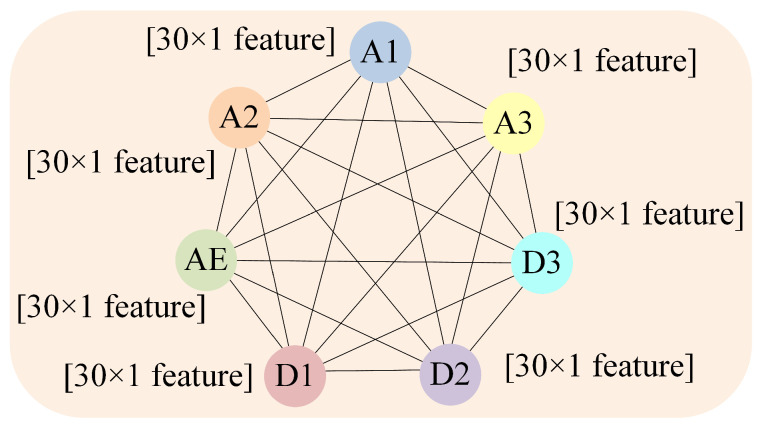
Graph-structured data for each cut in PHM2010.

To apply the proposed method to the PHM2010 dataset, signal feature extraction is performed on multi-sensor data, followed by the construction of a graph. Each cut from experiments C1 to C6 is converted into a single graph. A graph consists of seven nodes, each representing one of seven sensors: three for cutting forces, three for vibration signals, and one for acoustic emission signals. Feature vectors extracted from multi-sensor data are used to embed corresponding nodes. Nodes in each graph are fully connected (edges between nodes can be deleted, directed, or weighted according to further research to better reflect real-world conditions; in this study, we focus on undirected, fully connected graphs with a weight of 1 for all edges), forming a complete graph structure, as illustrated in Figure 8.

To begin with, the ULDTM is trained on unlabeled data to improve its capability to capture latent features from unlabeled data. Specifically, the cutting data from experiments C2 (cuts 1–289), C3 (cuts 1–238), and C5 (cuts 1–238) are used to construct graphs that are then input into the GNN. (The selection of the number of samples for the unlabeled datasets from C2, C3, and C5 is based on an estimation of the cutting samples before tool wear failure, and this number is flexible and can be adjusted according to research requirements.) The GNN output vectors’ L2 norms are calculated, then a monotonicity loss is calculated and backpropagated. During each training epoch, the parameters of the GNN of the ULDTM are saved. The parameters from the epoch with the smallest monotonicity loss are chosen as the initial parameters for the GNN of the LDTM.

In this paper, 0.16 mm of tool wear is used to define the tool failure threshold. Tool RUL labels are calculated as the total number of cuts from the start of the cutting process to the tool failure threshold minus the current cut number. For example, if the first 306 cuts from experiment C1 are chosen, the tool RUL label for the first cut is 305, the second cut is 304 and so on, with the 305th cut labeled 1 and the 306th cut labeled 0. Based on the tool failure threshold, 306 cuts are chosen from experiment C1 and 278 cuts from experiment C4. These chosen cuts form a training dataset of 584 samples (306 from C1 and 278 from C4). Additionally, the first 238 cuts from experiment C6 are chosen to form a validation dataset of 238 samples. The structure of the PHM2010 training/validation dataset is shown in Table 4.

### 3.2. Experimental Design of JUST_T05

Additionally, the JUST_T05 dataset is used as a supplementary experiment. JUST_T05 is a milling dataset built in a local laboratory. The cutting tool is a four-flute milling cutter. Similar to the PHM2010 dataset (Figure 7), the JUST_T05 dataset adopts a multi-sensor signal acquisition approach. In this paper, seven sensor signals are used to predict tool RUL. Three force sensors collect force signals in X, Y, and Z directions (N), three acceleration sensors collect vibration signals in X, Y, and Z directions (g), and one AE sensor collects the acoustic emission signal (dB). All cutting data are collected under the same working conditions.

The dataset is structured into three parts:

T_1: Unlabeled data used for training by the ULDTM.

T_2: Labeled data, containing 35 cuts, used for the LDTM as the training dataset.

T_3: Labeled data, containing 35 cuts, used for the LDTM as the validation dataset to predict tool RUL. Each cut in T_3 is structured as a graph, similar to the one shown in Figure 8.

## 4. Results and Discussion

### 4.1. PHM2010

**Figure 9:**
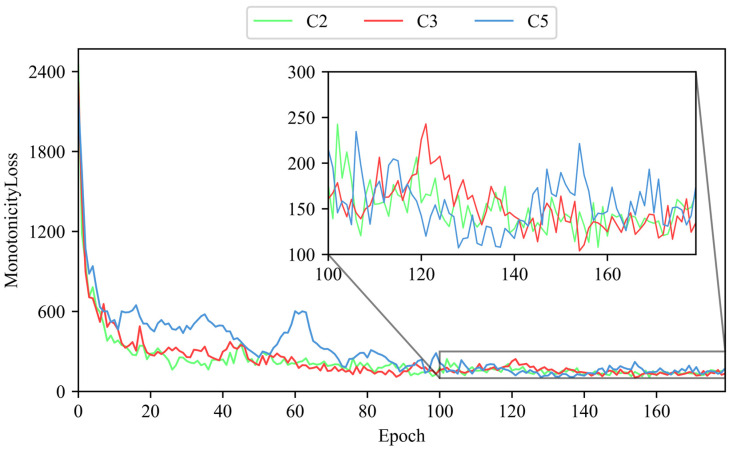
The monotonicity loss output by ULDTM for C2, C3, C5.

To evaluate the capability of the ULDTM in capturing latent features from tool cutting data, this paper uses the unlabeled datasets from C2, C3, and C5. A smaller monotonicity loss indicates that the ULDTM is more able to capture latent features which evolve as the number of cuts increases. Specifically, the ULDTM separately receives 289 graphs from dataset C2, 238 graphs from C3, and 238 graphs from C5. The monotonicity loss output by each training epoch for datasets C2, C3, and C5 is recorded. The monotonicity loss output by the ULDTM for C2, C3, and C5 is shown in Figure 9.

The results indicate that as the number of epochs increases, the monotonicity loss gradually decreases and eventually converges. This trend shows that guided by the custom criterion, the model can learn to extract latent features from unlabeled data.

To improve the accuracy and generalization capability of tool RUL prediction LDTM, this paper uses the ULDTM trained on unlabeled datasets. The parameters of the GNN part are first trained on unlabeled data, and these trained parameters are then used as the initial parameters for the GNN part of the LDTM, accomplishing transfer learning. This method considerably reduces the RMSE (root mean square error) of the LDTM calculated between predicted tool RUL and actual labels.

**Figure 10:**
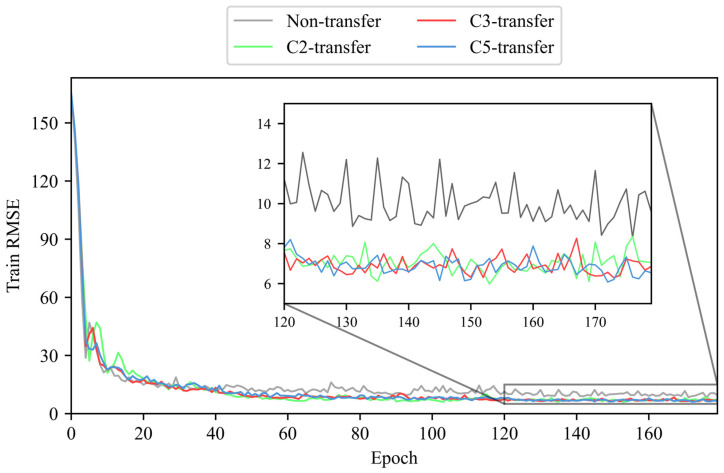
The RMSE of training phase without/with unlabeled data enhancement (no unlabeled data enhancement and enhancement from unlabeled dataset C2, C3, or C5).

**Figure 11:**
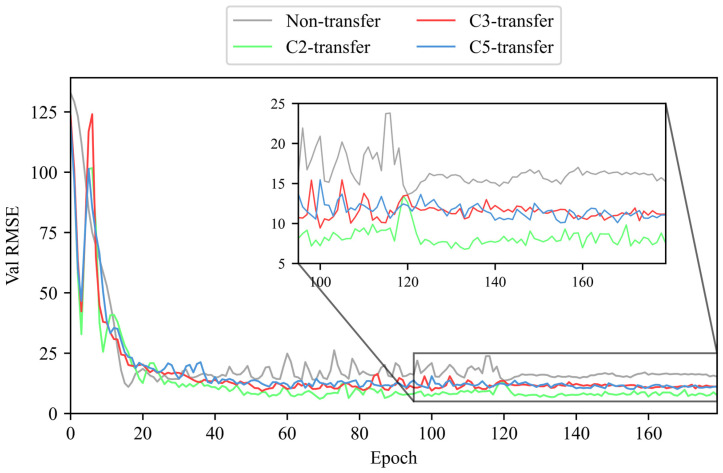
The RMSE of validation phase without/with unlabeled dataset enhancement (no unlabeled data enhancement and enhancement from unlabeled dataset C2, C3, or C5).

We use the LDTM to train and validate tool RUL prediction, respectively, on the training dataset (C1 and C4) and validation dataset (C6) [38]. This paper compares the performance of the LDTM under four conditions: without transfer learning (non-transfer) and with transfer learning enhancement from unlabeled datasets C2 (C2-transfer, etc.), C3, and C5. The RMSE is recorded during both training and validation phases. To ensure the reliability and credibility of the experimental results, each result presented is the median of multiple trials. The RMSE values of training and validation phases without/with unlabeled dataset enhancement are shown in Figure 10 and Figure 11.

The RMSE is calculated by the formula given below:

(9) $$\mathrm{RMSE}=\sqrt{\frac{1}{n}\sum_{i=1}^{n}{\left({\hat{y}}_{i}-y_{i}\right)}^{2}}$$

where $y=\{y_{1},y_{2},\ldots ,y_{n}\}$ represents the real tool RUL and $\hat{y}=\{{\hat{y}}_{1},{\hat{y}}_{2},\ldots ,{\hat{y}}_{n}\}$ represents the predicted tool RUL.

According to the results shown above, it is evident that the use of transfer learning leads to a more stable convergence of RMSE, as well as oscillation reduction and a smaller convergence value during both training and validation of tool RUL prediction. This indicates that the proposed method can effectively utilize unlabeled data to enhance the training and validation of labeled data tool RUL prediction.

**Figure 12:**
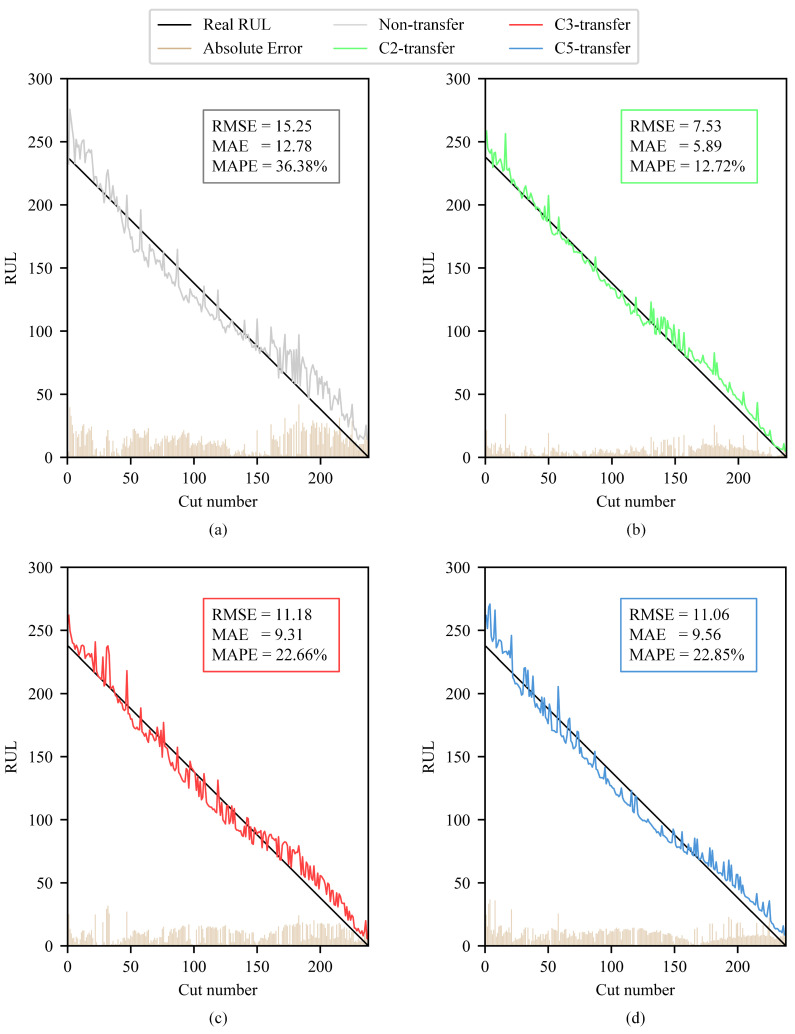
Comparison of the real tool RUL (labels), the tool RUL prediction without/with transfer learning (non-transfer, C2-transfer, etc.), and the absolute error between labels and tool RUL prediction on the validation dataset C6.

A comparison chart is drawn to compare the real RUL (labels), the RUL prediction without/with transfer learning, and the absolute error between labels and tool RUL prediction on the validation dataset C6. The chart clearly illustrates that the RUL prediction using transfer learning is more accurate than that without transfer learning, with a smaller RMSE and more generalization capability. The comparison of tool RUL prediction without/with unlabeled data enhancement is shown in Figure 12.

In addition to the formula given above for calculating RMSE, the formulas for calculating MAE (mean absolute error) and MAPE (mean absolute percentage error) are as follows (because the real tool RUL of MAPE cannot appear as 0, the term with real tool RUL 0 is skipped):

(10) $$\mathrm{MAE}=\frac{1}{n}\sum_{i=1}^{n}|{\hat{y}}_{i}-y_{i}|$$

(11) $$\mathrm{MAPE}=\frac{100\%}{n}\sum_{i=1}^{n}\left|\frac{{\hat{y}}_{i}-y_{i}}{y_{i}}\right|$$

This paper conducts additional experiments to study the broad applicability of the proposed method for using unlabeled data to improve tool RUL prediction on labeled data, as well as to demonstrate that a GNN can extract more useful information from multi-sensor data, thereby improving feature extraction quality. This paper replicates the proposed method in tool RUL prediction experiments using a convolutional neural network (CNN), multi-layer perceptron (MLP), transformer (Trans.), generative adversarial network (GAN), autoencoder (AE), and long short-term memory (LSTM). These models are also trained under two conditions: without transfer learning (no unlabeled data enhancement) and with transfer learning (unlabeled data enhancement). In these experiments, this paper continues to use C1 + C4 as the training dataset and C6 as the validation dataset. Model performance is evaluated by calculating the RMSE, MAE, and MAPE between the labels and the predicted tool RUL of C6.

**Figure 13:**
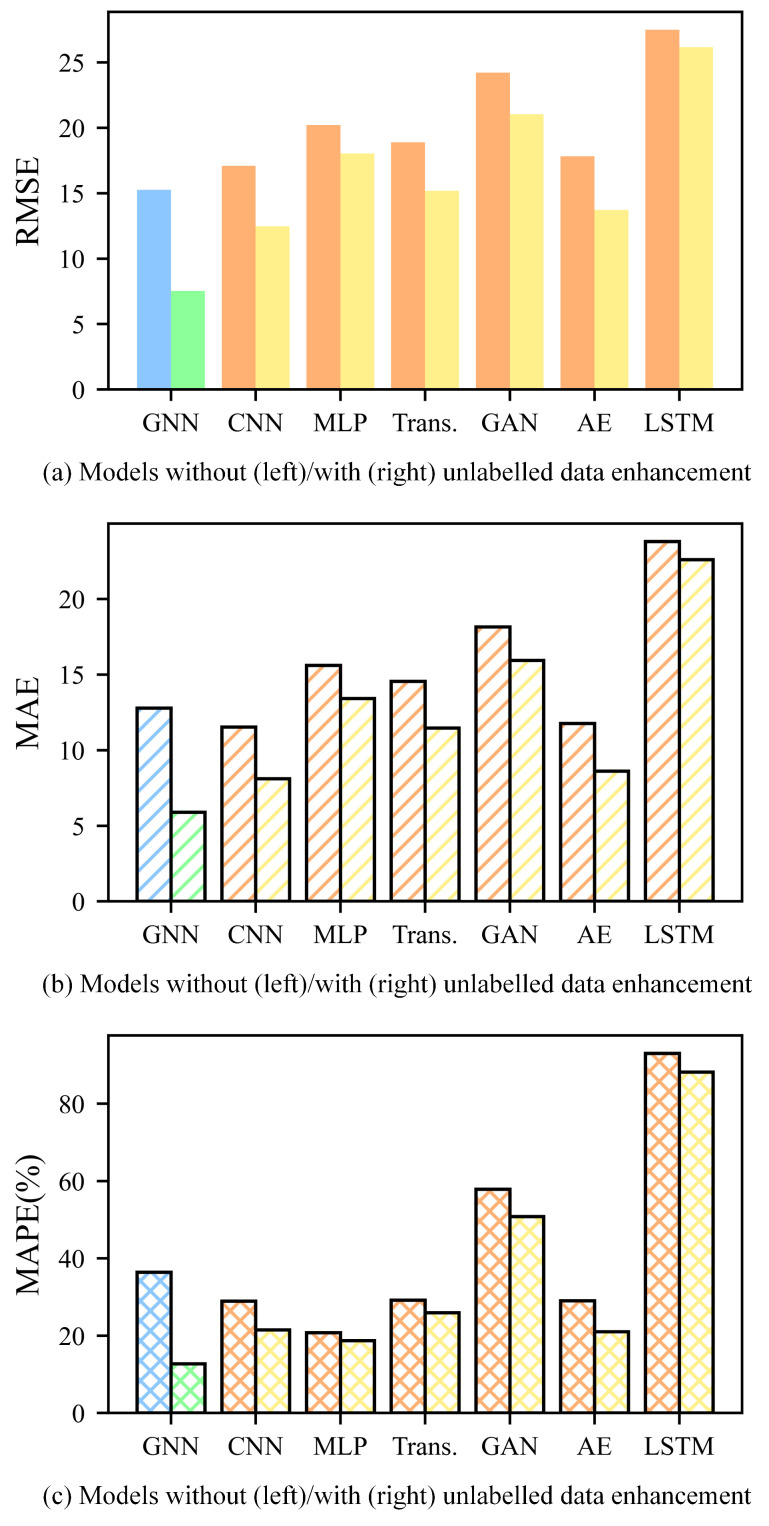
The RMSE, MAE, and MAPE of tool RUL prediction for models using C1 + C4 as the training dataset and C6 as the validation dataset. (The indicators are calculated based on the labels and predicted tool RUL of C6. As is shown in the figure, the indicators are smaller with unlabeled data enhancement across all these models).

The experimental results, as illustrated in the figure below, show that models achieve much smaller indicators (RMSE, MAE, and MAPE) in tool RUL prediction when using unlabeled data enhancement (the best result among C2-transfer, C3-transfer, and C5-transfer) than no unlabeled data enhancement (non-transfer). This shows that the proposed method improves the tool RUL prediction accuracy and generalization capability of the models. Moreover, the GNN outperforms other models in feature extraction from multi-sensor data, with smaller indicators under all conditions. In conclusion, the proposed method for unlabeled data enhancement not only improves the tool RUL prediction accuracy but also enhances the model’s generalization capability, confirming the effectiveness and broad applicability of the proposed method. The RMSE, MAE, and MAPE of tool RUL prediction for these models are shown in Figure 13.

### 4.2. JUST_T05

**Figure 14:**
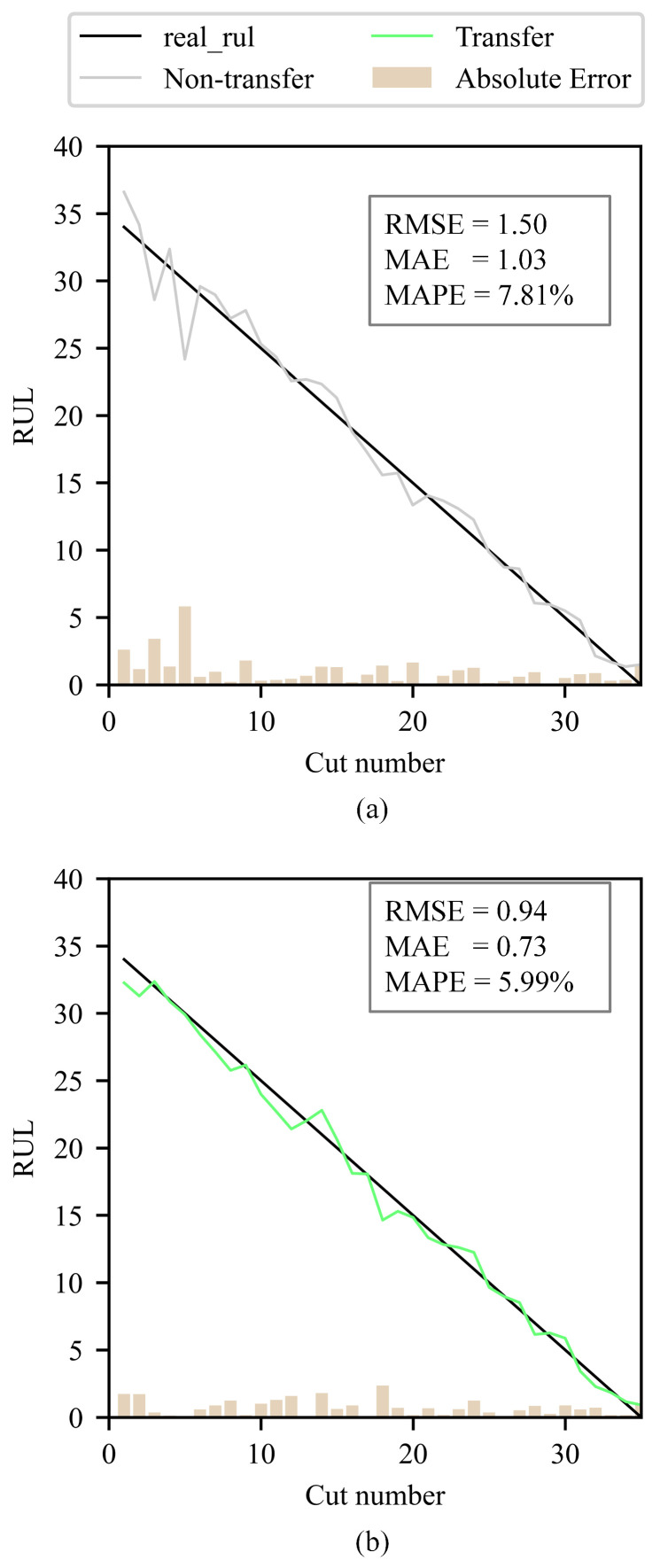
Comparison of the real tool RUL (labels), the tool RUL prediction without/with transfer learning (non-transfer, transfer), and the absolute error between labels and tool RUL prediction on the validation dataset T_3.

To verify the broad applicability of the proposed unlabeled data enhancement method for tool RUL prediction, this paper conducts additional experiments using the self-built dataset JUST_T05. In these experiments, T_1 is used to train the ULDTM. T_2 is used as the training dataset and T_3 as the validation dataset to predict tool RUL using the LDTM. The comparison of tool RUL prediction without/with unlabeled data enhancement on JUST_T05 is shown in Figure 14.

**Figure 15:**
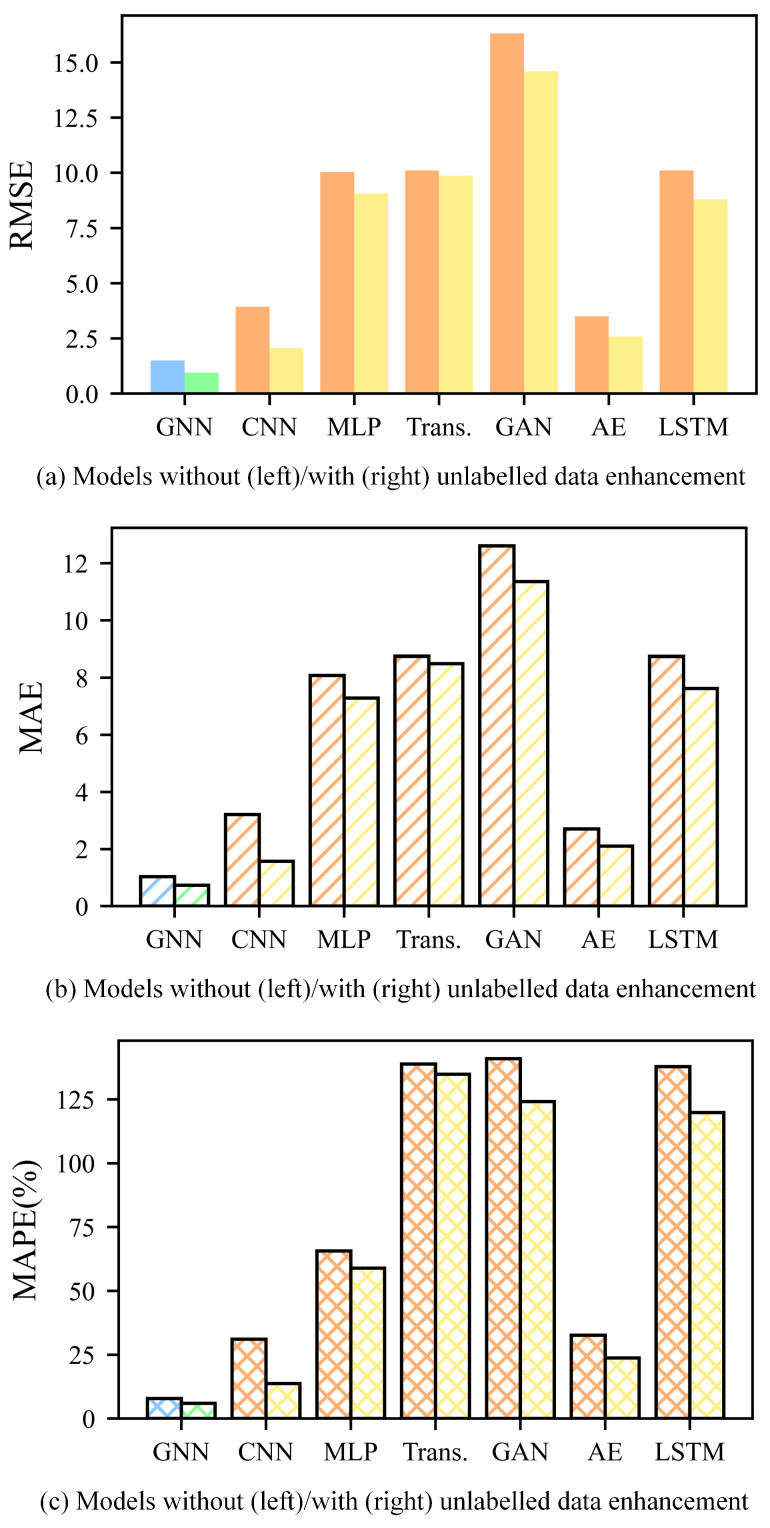
The RMSE, MAE, and MAPE of tool RUL prediction for models using T_2 as the training dataset and T_3 as the validation dataset. (The indicators are calculated based on the labels and predicted tool RUL of T_3. As is shown in the figure, the indicators are smaller with unlabeled data enhancement across all these models).

This paper focuses on enhancing tool RUL prediction accuracy and the generalization capability of models using the proposed unlabeled data enhancement method. Therefore, we applied the proposed method to other models to study its wide applicability. This paper replicates the proposed method on JUST_T05 using CNN, MLP, Trans., GAN, AE, LSTM. These models are also trained under two conditions: without transfer learning (no unlabeled data enhancement) and with transfer learning (unlabeled data enhancement). In these experiments, T_1 is used to train the ULDTM. T_2 is used as the training dataset and T_3 as the validation dataset. Model performance is evaluated by calculating the RMSE, MAE, and MAPE between the labels and the predicted tool RUL of T_3. As is shown in Figure 15, the proposed method also achieves smaller indicators on the self-built dataset JUST_T05. This demonstrates that the proposed method can effectively use unlabeled data, thereby improving the tool RUL prediction accuracy and generalization capability of the models.

## 5. Conclusions

This paper proposes a method for an unlabeled-data-enhanced tool remaining useful life prediction method based on a graph neural network. Compared with the tool RUL prediction model without unlabeled data enhancement, the model using unlabeled data enhancement achieves significant performance improvement. On the PHM2010 validation dataset, the RMSE of the tool RUL prediction model with unlabeled data enhancement is reduced by up to 50.6%, the MAE is reduced by up to 53.9%, and the MAPE is reduced by up to 23.66%. On the JUST_T05 validation dataset, the RMSE of the tool RUL prediction model with unlabeled data enhancement is reduced by up to 37.3%, the MAE is reduced by up to 29.1%, and the MAPE is reduced by up to 1.82%.

This method presents an innovative way for using unlabeled data to enhance tool RUL prediction on labeled data. Moreover, this paper uses a GNN to extract more useful information from multi-sensor data, thereby improving unlabeled data enhancement. The experimental results show that the proposed method improves the tool RUL prediction accuracy and generalization capability of the models. This paper offers a novel perspective for other researchers on utilizing unlabeled data and proves the applicability of GNNs in the field of unlabeled-data-enhanced tool RUL prediction.

In this paper, the graph-structured data is built as an undirected, fully connected graph with a weight of 1. In future research, some edges can be deleted, a directed graph constructed, and weights added to better reflect real-world conditions. Also, the custom monotonicity loss function in this paper can be replaced with other criteria (or does not need to be replaced), such as the coefficient of determination and trend strength, etc., to describe latent features that change when the number of cuts increases.

## Figures and Tables

**Table 1:** Time-domain characteristics.

Time-Domain Characteristics	Formula
Peak value	$y_{1}=\max(|x(i)|)$
Form factor	$y_{2}=\frac{\sqrt{\frac{1}{N}\sum_{i=1}^{N}x{\left(i\right)}^{2}}}{\frac{1}{N}\sum_{i=1}^{N}|x(i)|}$
Clearance factor	$y_{3}=\frac{y_{1}}{{\left(\frac{1}{N}\sum_{i=1}^{N}\sqrt{\left|x(i)\right|}\right)}^{2}}$
Shape factor	$y_{4}=\frac{\max(x(i))-\min(x(i))}{\sqrt{\frac{1}{N}\sum_{i=1}^{N}x{\left(i\right)}^{2}}}$

**Table 2:** Frequency-domain characteristics.

Frequency-Domain Characteristics	Formula
Mean value	$F_{1}=\frac{1}{K}\sum_{k=1}^{K}s(k)$
Centroid frequency	$F_{2}=\frac{\sum_{k=1}^{K}s(k)f_{k}}{\sum_{k=1}^{K}s(k)}$
Variance of frequency	$F_{3}=\sqrt{\frac{\sum_{k=1}^{K}s(k){\left(f_{k}-F_{2}\right)}^{2}}{K}}$
Frequency spread	$F_{4}=\frac{\sum_{k=1}^{K}s(k)\sqrt{\left|f_{k}-F_{2}\right|}}{K\sqrt{F_{3}}}$
Fourth moment of frequency	$F_{5}=\sqrt{\frac{\sum_{k=1}^{K}s(k)f_{k}^{4}}{\sum_{k=1}^{K}s(k)f_{k}^{2}}}$
Form factor	$F_{6}=\frac{\sum_{k=1}^{K}s(k)f_{k}^{2}}{\sqrt{\sum_{k=1}^{K}s(k)\sum_{k=1}^{K}s(k)f_{k}^{4}}}$
Coefficient of skewness	$F_{7}=\frac{F_{3}}{F_{2}}$
Third moment of the mean	$F_{8}=\frac{\sum_{k=1}^{K}s(k){\left(f_{k}-F_{2}\right)}^{3}}{KF_{3}^{3}}$
Fourth moment of the mean	$F_{9}=\frac{\sum_{k=1}^{K}s(k){\left(f_{k}-F_{2}\right)}^{4}}{KF_{3}^{4}}$
Energy concentration factor	$F_{10}=\frac{{\left(\sum_{k=1}^{K}s(k)f_{k}^{2}\right)}^{2}}{\sum_{k=1}^{K}s(k)f_{k}^{4}}$

**Table 3:** Structure of PHM2010 dataset.

	Unlabeled			Labeled		
Experiment number	C2	C3	C5	C1	C4	C6
Number of cuts	315	315	315	315	315	315
Number of sensors	7	7	7	7	7	7
Number of signals	7	7	7	7	7	7

**Table 4:** Structure of PHM2010 training/validation dataset.

PHM2010 Dataset	Chosen Samples	Description
C2	289	ULDTM training dataset
C3	238	ULDTM training dataset
C5	238	ULDTM training dataset
C1	306	LDTM training dataset_1
C4	278	LDTM training dataset_2
C6	238	LDTM validation dataset

## Data Availability

Data are contained within the article.
